# Characterization of brusatol self-microemulsifying drug delivery system and its therapeutic effect against dextran sodium sulfate-induced ulcerative colitis in mice

**DOI:** 10.1080/10717544.2017.1384521

**Published:** 2017-10-27

**Authors:** Jiangtao Zhou, Lihua Tan, Jianhui Xie, Zhengquan Lai, Yanfeng Huang, Chang Qu, Dandan Luo, Zhixiu Lin, Ping Huang, Ziren Su, Youliang Xie

**Affiliations:** a School of Pharmaceutical Sciences, Guangzhou University of Chinese Medicine, Guangzhou, PR China;; b Mathematical Engineering Academy of Chinese Medicine, Guangzhou University of Chinese Medicine, Guangzhou, PR China;; c Guangdong Provincial Key Laboratory of Clinical Research on Traditional Chinese Medicine Syndrome, The Second Affiliated Hospital, Guangzhou University of Chinese Medicine, Guangzhou, PR China;; d School of Chinese Medicine, Faculty of Medicine, The Chinese University of Hong Kong, Hong Kong, PR China

**Keywords:** Brusatol, self-microemulsifying drug delivery system, *in vitro* and *in vivo* evaluation, anti-colitis activity, anti-inflammation

## Abstract

Brusatol (BR) is one of the main bioactive components derived from *Brucea javanica*, a medicinal herb historically used in the treatment of dysenteric disorders (also known as ulcerative colitis(UC)). Due to its poor aqueous solubility, a novel brusatol self-microemulsifying drug delivery system (BR-SMEDDS) nanoformulation with smaller size, higher negative zeta potential and drug content, and excellent stability was developed. The appearance of BR-SMEDDS remained clear and transparent, and transmission electron microscopy showed microemulsion droplets to be spherical with homogeneous distribution. Pharmacokinetic parameters indicated that oral bioavailability was greatly improved by BR-SMEDDS as compared with aqueous suspension. Meanwhile, the anti-colitis activity of BR-SMEDDS was evaluated on dextran sodium sulfate (DSS)-induced colitis mice model. The result illustrated that the nano-formation significantly reduced the body weight loss, recovered colon length, decreased disease activity index and microscopic score, regulated immune-inflammatory cytokines, diminished oxidative stress and repressed the colonic expression of myeloid differentiation factor 88 (MyD88), toll-like receptor 4 (TLR4) and nuclear factor kappa B p65 (NF-κB p65) proteins. Our findings demonstrated for the first time that BR could effectively attenuate colonic inflammation in mice, at least partially, via favorable regulation of anti-oxidative and anti-inflammatory status and inhibition of the TLR4-linked NF-κB signaling pathway. The BR nano-formulation was superior to BR suspension and sulphasalazine, in treating experimental UC, and exhibited similar effect with azathioprine, with much smaller dosage. The enhanced anti-UC effect of BR might be intimately associated with the improved pharmacokinetic property by SMEDDS. The developed nano-delivery system might thus be a promising candidate for colitis treatment.

## Introduction

Ulcerative colitis (UC), one type of inflammatory bowel disease, is a chronic, relapsing gastrointestinal disorder characterized by intestine and colon inflammation, acute pain, vomiting, and diarrhea (Lombardi et al., 2011; Chen et al., 2015a). In recent years, due to a series of complicated factors, the incidence of UC presented a rising trend around the world (Yun et al., 2009), and it has been incorporated into the ranks of modern refractory diseases by World Health Organization (Bu, 2009). At present, common therapeutic agents for UC mainly include aminosalicylates, steroid hormone, immunosuppressive agents and antibiotics, most of which could relieve symptoms to some extent. However, these therapies have varying side effects (Liu & Wang, 2011; Fang et al., 2013). Therefore, it is imperative to discover potentially effective alternative for the treatment of UC.

In recent years, herbal medicines or natural compounds isolated from medicinal herbs have been shown to have beneficial effects on various inflammatory diseases including UC. *Brucea javanica* (L.) Merr (*Simaroubaceae*) is an important medicinal herb used in traditional folk medicine for treating various inflammatory disorders including dysentery, which is consistent with UC nowadays on the clinical symptoms (Han, 2014). Indeed, our previous study has shown that *Brucea javanica* oil emulsion possessed appreciable anti-inflammatory effect against murine experimental UC induced by dextran sodium sulfate (Huang et al., 2017). *Brucea javanica* oil and its medicinal preparation have also been demonstrated to be clinically effective in the treatment of UC through retention enema (Mao & Li, 2002; Sheng, 2012). Besides, *Brucea javanica* a medicinal herbal compound was also observed to exert pronounced therapeutic effect against trinitrobenzene sulfonic acid which induced experimental colitis in rats (Lei, 2013).

However, the active ingredients and the mechanism underlying these effects have not been investigated. Brusatol (BR), one of the major active quassinoids of *Brucea javanica*, was found to exhibit diverse bioactivities including antimalarial, antineoplastic, anthelmintic and hypoglycemic activities (Noorshahida et al., 2009; Zhang et al., 2013; Turpaev & Welsh, 2015). Furthermore, BR was reported to be a potent anti-inflammatory agent by inhibition of protein synthesis (Hall et al., 1983; Tang et al., 2014). In our prior trial, BR exhibited appreciable protective effect against experimental UC. Nevertheless, the low aqueous solubility and rapid first-pass metabolism after oral administration have severely limited its potential medicinal application. In order to improve the dissolution and bioavailability of BR, we focused our attention on a nanoformulation named self-microemulsifying drug delivery system (SMEDDS).

Over the past decades, SMEDDS has been developed into a universal pharmaceutical means to improve oral bioavailability for insoluble lipophilic drugs delivery. SMEDDS is composed of oils, surfactants, cosurfactants and drugs (poor solubility ones) mixtures, which spontaneously form oil-in-water (O/W) microemulsion with nanometric droplet size in aqueous medium or gastrointestinal fluids under mild agitation (Guan et al., 2016). Recently, many insoluble lipophilic drugs have been developed for SMEDDS formulations, such as curcumin (Cui et al., 2009; Setthacheewakul et al., 2010; Wu et al., 2010; Zhang et al., 2012), resveratrol (Bolko et al., 2014; Chen et al., 2015c), docetaxel (Chaurasiya et al., 2012; Seo et al., 2013), naproxen (Vrečer, 2014) and berberine hydrochloride (Zhu et al., 2013). Drug delivery advantages offered by SMEDDS include not only increasing drug solubilization, but also improving both release and absorption properties through enhancing permeation across the inflamed mucosal tissues (Li et al., 2015). The smaller particle diameter provides a large interfacial surface area for drug absorption in the gastrointestinal tract. Furthermore, the lipids play an essential role in protecting the drug against enzymatic hydrolysis, and surfactant-induced membrane fluidity and subsequent permeability changes, which bring about potentially enhancing absorption (Sha et al., 2005; Li et al., 2015).

The aim of the present study was to develop a nanoformulation of SMEDDS containing BR to increase its solubility and bioavailability, and enhance its anti-colitis activity. The BR-SMEDDS formulation was characterized by evaluating its physicochemical characterizations including droplet size, polydispersity index (PDI), morphology, zeta potential, drug encapsulation efficiency, stability, as well as pharmacokinetics. Meanwhile, the formulation was tested *in vivo* for the anti-colitis activity and potential underlying mechanism in DSS-induced UC mice. Sulphasalazine (SASP) and azathioprine (AZA), two kinds of positive drugs currently used in the treatment of UC, served for comparisons.

## Materials and methods

### Chemical and reagents

Kolliphor® HS15 (PEG 660-12-hydroxystearate, BASF, Ludwigshafen, Germany), and PEG 400 were obtained from the Tianjin Zhiyuan Chemical Reagent Co., Ltd (Tianjin, China). Mediumchain triglyceride (C8, MCT) was purchased from Guangdong Mingkang Flavors & Fragrances Co., Ltd. (Guangzhou, Guangdong, China). DSS was the product of MP Biomedicals (molecular weight: 36000-50000, MP Biomedicals, Canada). SASP was purchased from Shanghai Fuda Pharmaceutical Co., Ltd. (Shanghai, China). AZA was purchased from Aspen Pharmacare Australia Pty Ltd. (Leonards, New South Wales, Australia). Pure water was supplied by A.S. Watson Group Ltd. (Hong Kong, China). All other ingredients, reagents and solvents were of analytical grade.

### Isolation and identification of BR

The seeds of *Brucea javanica* were provided by Ming Xing Pharmaceutical Co. Ltd. (Guangzhou, Guangdong, China) and extracted twice with 95% EtOH under heating for 2 h. The solvent was concentrated to give a crude extract, followed by suspending in H_2_O. The aqueous layer was further extracted with EtOAc. The EtOAc layer was evaporated under vacuum to afford extracts and subjected to silica gel column chromatography eluted with a gradient of CH_2_Cl_2_:MeOH (100:0–100:20). The CH_2_Cl_2_:MeOH (100:1) eluate was evaporated to yield a residue, which was further purified by repeated recrystallization to obtain a white powder. The structure of the white powder was elucidated by nuclear magnetic resonance (NMR). The purity of this compound was above 95% through high-performance liquid chromatography (HPLC) (Shimadzu HPLC system, Kyoto, Japan). The chromatographic separation was performed on a C_12_ column (Phenomenex Synergi 4 µm MAX-RP 80 A, 4.6 × 150 mm, USA) with the mobile phase composed of acetonitrile and aqueous solution (55:45, vol/vol) at a flow rate of 1.0 mL/min. The wavelength of detection was 277 nm.

### Preparation of BR-SMEDDS formulation

In our preliminary test, pseudo-ternary phase diagram was adopted to optimize the composition of BR-SMEDDS, and the best formulation was found to be the MCT/HS 15/PEG 400 combinations. MCT, HS 15 and PEG 400 were selected as oil, surfactant and co-surfactant, respectively. Blank SMEDDS formulation was prepared by mixing the HS 15 and PEG 400 in a water bath at 37 °C, and MCT was added with magnetic stirring at 300 rpm for 10 min at 30 °C at the ratio of 10:40:20 (w/w/w). At last, the prescription amount of BR was accurately weighed into the mixtures. After gentle magnetic stirring (50 rpm), the transparent SMEDDS formulation was formed (containing 20 mg/g BR). The mixture was stored at room temperature until used. Before the *in vitro* and *in vivo* test, SMEDDS were prepared by diluting with distilled water. Blank SMEDDS formulation was prepared following the same method above mentioned without BR.

### Characterization of BR formulation

#### Droplet size, polydispersity index, and zeta-potential determination

The SMEDDS formulation was diluted 10 times using distilled water and stirred at 50 rpm using a rotating magnetic stirring apparatus. The droplet size, polydispersity index (PDI) and zeta-potential were determined using a Malvern Zetasizer Nano ZS90 (Malvern Instruments Co. Ltd., Malvern, UK). The means of particle diameter, PDI and zeta potential were obtained from three batches of samples.

#### Morphology measurements

The morphology of BR-SMEDDS was morphologically observed by transmission electron microscopy (TEM) (JEM-1400, JEOL, Tokyo, Japan). After dilution with distilled water at the ratio of 1:500, a sample drop was placed on a copper grid and dyed with phosphotungstic acid (2%) for 30 s. The grid was dried at room temperature to form a thin film and then observed under the electron microscope.

#### Drug encapsulation efficiency

The encapsulation efficiency of BR-SMEDDS was determined using a combined ultrafiltration-centrifugation technique according to the method described previously (Ma et al., 2013). BR-SMEDDS (0.1 g) was diluted with 10 mL distilled water and transferred to the upper chamber of the centrifuge tubes fitted with an ultrafilter (4 mL/10 kD Amicon® Ultra-4, Millipore, Billerica, MA). The tubes were centrifuged by a high-speed refrigerated centrifuge (TGL16M, Hunan, China) at 5000 rpm for 20 min. The supernatant was used for the concentration of encapsulated drug (*C_e_*), which was determined by HPLC. To determine the concentration of total drug (*C_t_*), 0.1 g BR-SMEDDS was dissolved in methanol, and the concentration of BR was measured by HPLC analysis. The chromatographic conditions were consistent with those of the identification procedure. The encapsulation efficiency (EE %) was calculated by Equation (1).

(1) $$\text{EE}\;\left(\text{\%}\right)\text{=}\frac{\mathrm{Ce}}{\mathrm{Ct}}\text{ × 100\%}$$

#### *Stability* in vitro

In order to explore whether the formulation was stable in the transition from stomach to intestine after oral administration, we incubated the BR-SMEDDS in simulated gastric fluid (HCl solution, pH 1.2) and intestinal fluid (PBS, pH 7.4) environments at 37 °C, respectively. Artificial gastric juice and intestinal juice were prepared according to the Chinese Pharmacopoeia (National Pharmacopoeia Committee, 2010). Then, the droplet size, PDI and zeta potential were determined as described above.

To investigate the physical stability,1 gBR-SMEDDS was diluted with 10 mL distilled water and centrifuged at 10000 rpm for 20 min at 25 °C. They were assumed to be physically stable provided that no signs of drug precipitation, droplets conjugation, creaming and phase separation were visually observed.

#### Bioavailability study

All experimental procedures involving the use of animals in this paper were reviewed and approved by the Ethics Review Committee for Experimental Animal Center of Guangzhou University of Chinese Medicine (Permit ID: 2013-0020), and all efforts were made to minimize possible suffering. All experiments were performed in accordance with the guidelines and regulations of Guangzhou University of Chinese Medicine.

Twelve male Sprague–Dawley rats (250 ± 20 g) were purchased from the Guangdong Medical Laboratory Animal Center (Guangzhou, China) and acclimatized in the laboratory for seven days before the pharmacokinetics experiment. All rats were fasted for 12 h but allowed free access to water. They were randomly divided into BR-SMEDDS group and BR-suspension group for oral administration (6 mg/kg BR in content). The dosage was selected based on previous report (Zhang et al., 2016b) and our preliminary experiment. To prepare the suspension, BR was weighed and then suspended in 0.5% (wt/vol) sodium carboxymethycellulose (CMC-Na) solution to the concentration of 0.6 mg/mL. Approximately 0.5 mL blood samples were collected into heparinized Eppendorf tubes at 0.083, 0.25, 0.5, 1.0, 1.5, 2.0, 2.5, 4.0, 6.0, 12 and 24 h from the eye socket vein following oral dosing. The plasma was separated from the whole blood by centrifugation at 5000 rpm for 10 min and stored at −20 °C for further analysis.

The method of plasma sample preparation was determined as previously described by Zhang et al. (2016b). Briefly, 100 µL plasma was mixed with 10 µL brucein D (200 ng/mL, internal standard) and 500 µL ethyl acetate in a vortex mixer for 3 min. The mixture was centrifuged at 6000 rpm for 10 min. Then, all supernatants were transferred into another clean tube and dried at 40 °C with a vacuum degree at −0.8 Mpa. The residue was re-dissolved with 50 µL methanol/water (50:50, vol/vol) by vortexing for 3 min. After centrifugation for 10 min, the supernatant was transferred to the autosampling vials for introduction into the LC-MS/MS system.

The analysis was performed with an Agilent 1260 Infinity HPLC system interfaced with an Agilent 6460 Triple Quad LC-MS/MS system with an electrospray ionization source. Separation was achieved on a C_18_ column (Symmetry® 3.5 µm, 2.1 × 100 mm, Waters, Ireland) and eluted on an isocratic mobile phase composed of methanol and 10 mM ammonium acetate containing 0.1% (vol/vol) formic acid (50:50, vol/vol) at a constant flow rate of 0.1 mL/min .

Pharmacokinetics parameters were calculated using the Drug and Statistics software (version 2.11, Mathematical Pharmacology Professional Committee of China, Shanghai, China). With HPLC-MS/MS detection, the standard curve of BR concentration in plasma was linear in the range of 5–200 ng/mL with a regression equation of Y= 669869 × C + 2068.4 (*r* ^2^ = 0.9994).

### Anti-colitis activity of BR-SMEDDS

#### Animals and experimental design

A total of 160 adult male BALB/c mice weighing 20–24 g, were purchased from the Experimental Animal Center of Guangdong Province. The animals were housed in a temperature-controlled (22–24 °C) room with a light/dark cycle of 12 h. All the animals were fed a standard laboratory chow and tap water was allowed *ad libitum*. After two weeks of acclimation, mice were randomly sorted into eight different experimental groups (20 mice per group). Acute ulcerative colitis was induced by adding DSS (dissolved in fresh distilled water) to the drinking water to a final concentration of 3% (wt/vol) for seven consecutive days. (1) Normal group received distilled water (Normal control group). (2) DSS-treated group received blank-SMEDDS diluted with distilled water (DSS group). (3) DSS-treated mice received 200 mg/kg sulfasalazine per day (SASP group). (4) DSS-treated mice received 13 mg/kg azathioprine per day (AZA group). (5) DSS-treated mice received 0.25 mg/kg BR per day (BRL-SMEDDS group). (6) DSS-treated mice received 0.5 mg/kg BR per day (BRM-SMEDDS group). (7) DSS-treated mice received 1 mg/kg BR per day (BRH-SMEDDS group). (8) DSS-treated mice received 1 mg/kg BR per day (BR-suspension group). The dose of BR was selected based on previous reports (Hall et al., 1983; Tang et al., 2014) and our pilot study. The powders of SASP and AZA were added to fresh distilled water and were made into suspending liquid. BR-SMEDDS was diluted with distilled water to the concentrations of 0.025, 0.05 and 0.1 mg/mL, respectively, before oral administration. BR-suspension was formulated by dispersing the compound in 0.5% CMC-Na solution to the concentration of 0.1 mg/mL. All the solutions were administered by intragastric gavage once a day for seven consecutive days (0.1 mL/10 g body weight).

#### Samples collection

Mice were sacrificed on day 8, and colons (from the ileocecal junction to the anal verge) were removed, free of adherent adipose tissue, rinsed with saline to remove fecal residue, and the lengths and weights were measured. Colons were collected for hematoxylin and eosin (H&E) staining, cytokine analyses and Western blot assay.

#### Disease activity index

Animal health status was recorded daily for weight changes, water/food consumption, fecal character, diarrhea, and rectal bleeding. The DAI was determined by a person who was not clear about the experimental protocol at the end of the experiment as described previously (Han et al., 2015). Briefly, body weight loss (0, none; 1, 1–5%; 2, 5–10%; 3, 10–20%; and 4, >20%), stool consistency (0, normal; 2, loose stools; and 4, diarrhea), and stool blood (0, no blood; 2, positive occult blood; and 4, gross bleeding) were recorded daily. Body weight loss was calculated as the percentage difference between the body weight on day 0 and that on the day the animal was weighed. These scores were added together and divided by three, resulting in DAIs ranging from 0 (healthy) to 4 (maximal colitis activity). The colonic length was measured in centimeter.

#### Histologic assessment of colons

The distal colorectum was fixed in 4% paraformaldehyde, embedded in paraffin and stained with H&E in accordance with the standard procedures for histological evaluation. The histological damage and colonic inflammation were observed microscopically using a DP73 light microscope (Olympus, Tokyo, Japan). Histological scores were determined as Li et al. (2014b) previously described.

#### Biochemical analysis of colons

Colonic tissues (10% wt/vol) were homogenized in PBS containing 1% Proteases Inhibitor Cocktail (Sigma-Aldrich, St Louis, MO) at 4 °C. After centrifugation, supernatants were collected. The concentrations of tumor necrosis factor-α (TNF-α), interferon-γ (IFN-γ), interleukin-1β (IL-1β), interleukin-6 (IL-6), interleukin-4 (IL-4), interleukin-10 (IL-10) and prostaglandin E_2_ (PGE_2_) in the colorectal tissues were quantified by enzyme-linked immune sorbent assays (NeoBioscience, China) according to the manufacturer's instructions. Cytokine content was determined as pg/mg or ng/mg of total colon protein. Colon homogenates were used for the determination of myeloperoxidase (MPO), malondialdehyde (MDA), superoxide dismutase (SOD) and glutathione peroxidase (GSH-Px) levels using commercial kits (Nanjing Jiancheng Bioengineering Institute, China). The amount of total protein concentration was measured by BCA assay (Nanjing Jiancheng Bioengineering Institute, China). The absorbance was measured using a multimode microplate reader (Multiskan GO 1510, Thermo Fisher Scientific, Vantaa, Finland).

#### Western blot analysis

Colorectal tissues were homogenized using RIPA buffer (50 mM Tris (pH 7.4), 150 mM NaCl, 1% Triton X-100, 1% sodium deoxycholate, 0.1% SDS, 10 mM NaF, 5 mM EDTA, 1 mM Na_3_VO_4_) with Protease Inhibitor Cocktail (1:100). After centrifugation, supernatants were collected. The protein content was estimated by using the Pierce^TM^ BCA Protein Assay Kit (Pierce, Thermo scientific, Rockford, IL). Proteins (10 μg) were electro-blotted on polyacrylamide gel-electrophoresis, and the separated proteins were transferred onto a PVDF membrane. Subsequently, the membrane was blocked with 5% skimmed milk in TBST for 1 h and then incubated at 4 °C overnight with the corresponding primary antibodies against MyD88, TLR4, NF-κB p65 and β-actin (internal control) (Santa Cruz Biotechnology, Santa Cruz, CA). The obtained chemiluminescence signals were quantified using Image J software (National Institutes of Health, Bethesda, MA).

### Statistical analysis

GraphPad Prism 5 (GraphPad Software, Inc.) software was used for statistics and plotting. Data were expressed as means ± standard deviation (SD). The difference in the results was assessed using Student’s t-test or one-way analysis of variance followed by Dunnett’s test of SPSS (version 13.0; SPSS Inc.) software. A significant difference was identified at *p* < .05 or .01.

## Results

### Structure elucidation of BR

**Figure 1.**
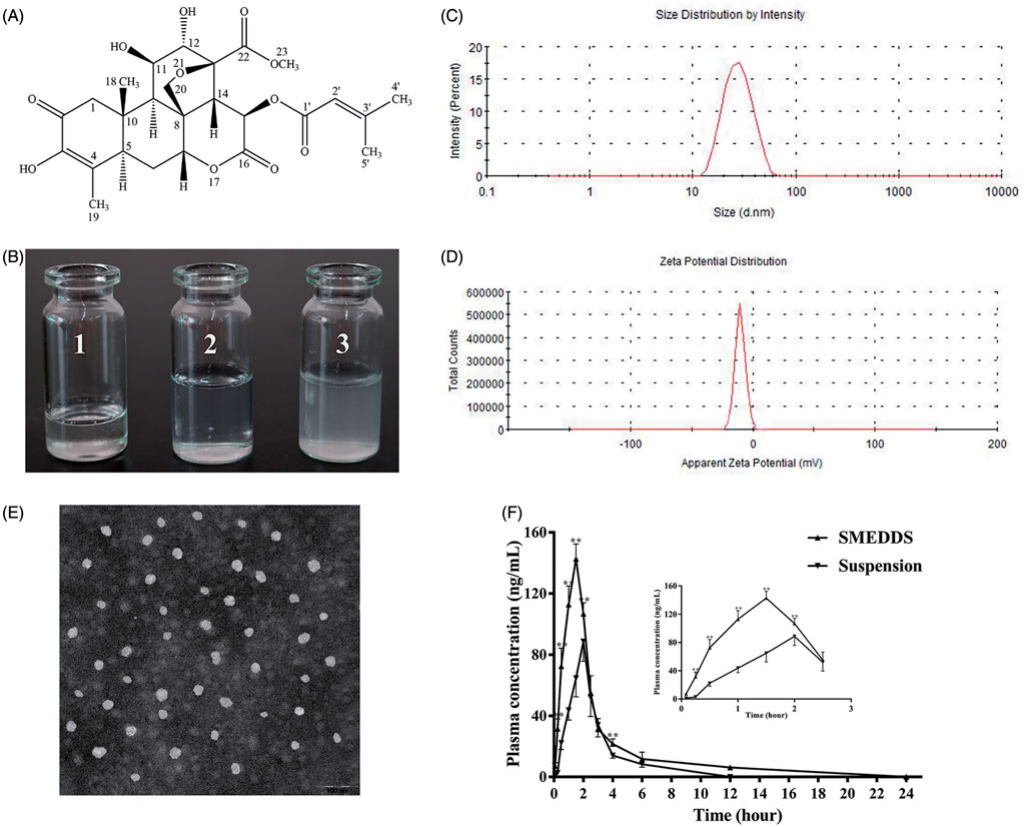
(A) The chemical structure of brusatol. (B) Appearance of BR-SMEDDS under different conditions: 1. Appearance of BR-SMEDDS at room temperature; 2. Appearance of BR-SMEDDS diluted 100-fold with distilled water; and 3. Appearance of BR-suspension. (C) Particle size and distribution of BR-SMEDDS. (D) Zeta potential of BR-SMEDDS. (E) TEM image of droplet BR-SMEDDS. (F) Plasma concentration-time profiles of rats after oral administration with BR-SMEDDS and BR-suspension. Data are expressed as mean ± SD (*n* = 6). *p < .05 and **p < .01 versus suspension group.

^1^H NMR (400 MHz, DMSO-d_6_) *δ* ppm: 2.63 (1H, s, H-1α), 3.28 (1H, d, *J* = 12.2 Hz, H-1β), 2.89 (1H, d, *J* = 12.8 Hz, H-5), 2.07 (1H, d, *J* = 2.0 Hz, H-6α), 1.71 (1H, m, H-6β), 4.92 (1H, s, H-7), 2.16 (1H, d, *J* = 3.2 Hz, H-9), 3.97 (1H, s, H-11), 4.10 (1H, s, H-12), 1.22 (3H, s, H-18), 1.72 (3H, s, H-19), 3.59 (1H, s, H-20a), 4.48 (1H, d, *J* = 7.2 Hz, H-20 b), 3.61 (3H, s, H-23), 5.62 (1H, s, H-2'), 2.10 (3H, s, H-4'), 1.90 (3H, s, H-5'), 7.86 (3H, s, 3-OH), ^13 ^C NMR (100 MHz, DMSO-d_6_) *δ* ppm: 49.2 (C-1), 193.2 (C-2), 144.7 (C-3), 128.8 (C-4), 41.5 (C-5), 29.1 (C-6), 83.0 (C-7), 41.0 (C-8), 40.6 (C-9), 45.2 (C-10), 71.9 (C-11), 75.0 (C-12), 81.7 (C-13), no assignable (C-14, C-15), 167.8 (C-16), 15.5 (C-18), 13.7 (C-19), 72.7 (C-20), 170.4 (C-22), 52.5 (C-23), 164.5 (C-1'), 115.3 (C-2'), 158.8 (C-3'), 20.4 (C-4'), 27.4 (C-5'). Its spectroscopy data were consistent with the previous literature (Harigaya et al., 2004). Hence, the white powder obtained was identified as BR, and its structure was given in Figure 1(A).

### Characterization of BR-SMEDDS formulation

#### Appearance and morphology of BR-SMEDDS

As shown in Figure 1(B), BR-SMEDDS was a transparent viscous liquid at room temperature. Diluted with 100-fold distilled water, BR-SMEDDS formed a clear and transparent microemulsion with bluish opalescence. Meanwhile, the same content of BR was dispersed in 0.5% (wt/vol) CMC-Na solution, which was white turbid liquid. Hence, it was suggested that BR-SMEDDS could significantly increase the solubility of BR in the water. The morphology of BR-SMEDDS was observed by TEM and the images are shown in Figure 1(E). The TEM result displayed that most microemulsion droplets were nearly spherical with a small size, and dispersed homogeneously in aqueous medium.

#### Droplet size, zeta potential and encapsulation efficiency

As shown in Figure 1(C,D), the mean droplet size of BR-SMEDDS was 25.97 ± 0.42 nmwith PDI of 0.084 ± 0.01 ±  (*n* = 3), and the average zeta potential of BR-SMEDDS was −11.33 ± 1.15 mV (*n* = 3). At the same time, the entrapment efficiency of BR in SMEDDS was detected to be 94.95 ± 2.03% (*n* = 3).

#### Stability studies

The appearance of BR-SMEDDS diluted with different mediums did not exhibit significant alternations. The average particle sizes of the formulation was kept almost constant. At the same time, low PDIs were maintained among the three different types of mediums. Nevertheless, it seemed that the zeta potential changed under the acidic conditions (Table 1). In addition, after 20 min of centrifugation, the microemulsion maintained a clear and transparent liquid with bluish opalescence.

**Table 1.** Effects of different pH mediums on the stability of BR-SMEDDS.

Medium	Droplet size (nm)^a^	PDI	Zeta potential (mV)
Distilled water	26.43 ± 0.18	0.08 ± 0.02	−11.07 ± 2.17
HCl (pH = 1.2)	26.29 ± 0.25	0.08 ± 0.00	−17.77 ± 1.55
PBS (pH = 7.4)	26.58 ± 0.18	0.08 ± 0.01	−11.42 ± 2.81

^a^ Data are expressed as mean ± SD (*n* = 3).

#### In vivo *pharmacokinetics studies*

The mean plasma concentration–time curve profiles after oral administration with BR-SMEDDS and BR suspension was presented in Figure 1(F), and the pharmacokinetics parameters acquired by the non-compartmental analysis were listed in Table 2. The result suggested that the *T*_max_ was advanced approximately 1.33-fold in BR-SMEDDS as compared to the BR-suspension. The half-life (*t*_1/2_) of BR-SMEDDS was prolonged about 2.32-fold as compared to that of BR suspension. Moreover, the peak concentration (*C*_max_) of SMEDDS containing BR was 142.70 ± 9.72 ng/mL, which was nearly 1.6 times larger than that of BR suspension. The mean residence time (MRT_0–24_) of SMEDDS was 3.72 ± 0.32 h, significantly greater than that of the suspension. The area under the concentration-time curves from 0 to 24 h (AUC_0–24 h_) of SMEDDS and suspension was 401.58 ± 43.03 and 213.38 ± 23.26 ng·h/mL, respectively, yielding a relative bioavailability of 188.20%. The above data suggested that in the same content, SMEDDS could effectively improve the pharmacokinetics parameters of BR and prolonged its circulation time within the body.

**Table 2.** Pharmacokinetics parameters of BR-SMEDDS and BR-suspension after oral administration in rats.

Parameters	Suspension	SMEDDS
AUC_0−24_ (ng·h/mL)	213.38 ± 23.26	401.58 ± 43.03**
*t*_1/2_ (h)	0.59 ± 0.07	1.37 ± 0.03**
*T*_max_ (h)	2.00 ± 0	1.50 ± 0
*C*_max_ (ng/mL)	88.61 ± 12.89	142.70 ± 9.72**
MRT_0–24_ (h)	2.76 ± 0.14	3.72 ± 0.32**
Relative bioavailability (%)	–	188.20%

Values are expressed as mean ± SD (*n* = 6).

* *p* < .05

** *p* < .01 versus suspension group.

### Anti-colitis activity of BR-SMEDDS

#### General observations

**Figure 2.**
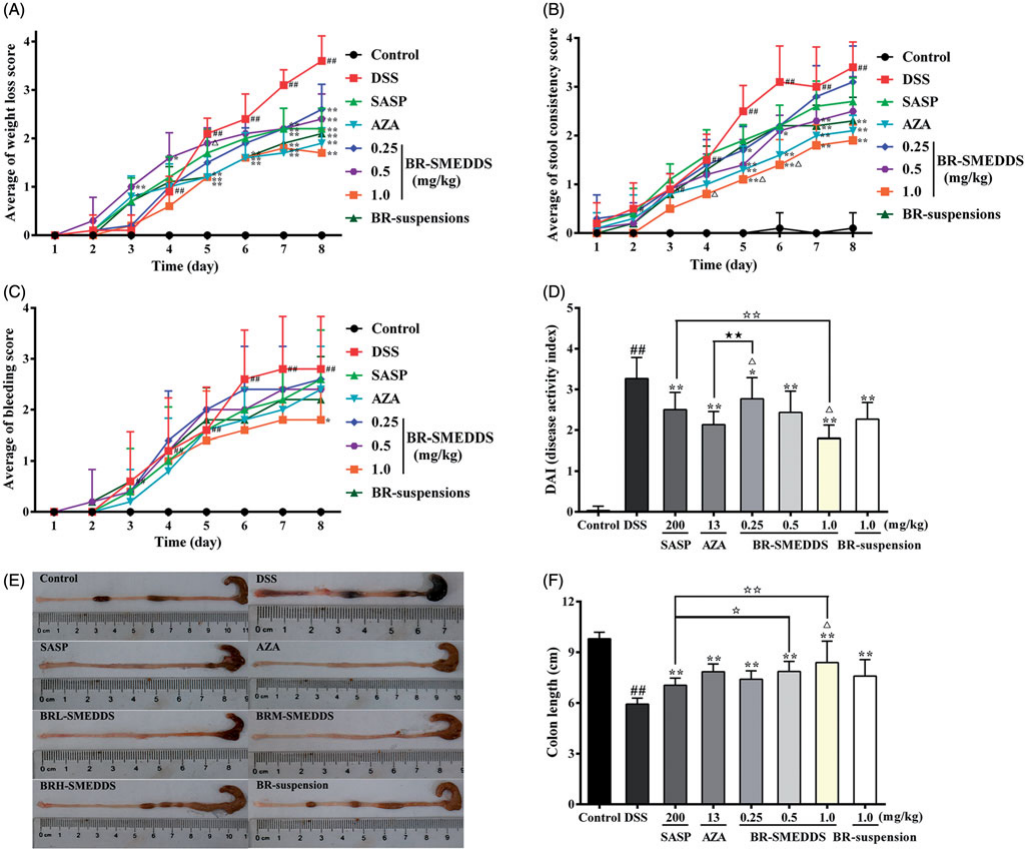
General performance of DSS-induced colitis in mice. (A) Weight loss score. (B) Stool consistency score. (C) Bleeding score. (D) Disease activity index. (E) Macroscopic appearances of colon tissues and (F) The lengths of colons. Mice receiving distilled water served as control. Data are presented as mean ± SD of 10 mice in each group. #*p* < .05 and ##*p* < .01 versus control group, *p < .05 and **p < .01 versus DSS group, ^△^ *p* < .05 and ^△△^ *p* < .01 versus BR-suspension group, ^⋆^ *p* < .05 and ^⋆⋆^ *p* < .01 versus SASP group, ^★^ *p* < .05 and ^★★^ *p* < .01 versus AZA group.

To assess the potential protective effect of BR-SMEDDS on DSS-induced colitis, mice were treated with three different doses of BR-SMEDDS, BR-suspension, and two positive drugs (SASP and AZA). As shown in Figure 2(A), DSS-treated colitis mice exhibited profound body weight loss since day 3, and did not recover at the end of the experiment. Whereas, BR-SMEDDS administration was shown to significantly attenuate the loss of body weight during the progression of colitis in mice. On the other hand, as shown in Figure 2(B,C), mice receiving DSS developed soft stools with mild traces of diarrhea since day 2. The severity of manifestations was progressively intensified towards the termination of experiment, where mice exhibited watery feces and gross bleeding on the anus site. However, oral administration with BR-SMEDDS resulted in different levels of suppression of diarrhea and protection against presence of occult blood. In summary, score of DAI increased remarkably after DSS intake as compared with the normal group (*p* < .01). However, treatment with BR-SMEDDS markedly attenuated the elevated DAI scores in mice induced by DSS in a dose-dependent manner (*p* < .05). As compared with the BR-suspension group, the effect of two types of positive drugs and BRM-SMEDDS (0.5 mg/kg) groups showed no statistically significant difference (*p* < .05). Nevertheless, the DAI score was much lower in the BRH-SMEDDS group (1 mg/kg) as compared with that of the BR-suspension group (1 mg/kg) (*p* < .05) and the SASP group (200 mg/kg, *p* < .01) (Figure 2(D)). The BRH-SMEDDS group showed analogous DAI score when compared with AZA group (13 mg/kg, *p* < .05). Besides, shortening of the colon length was also an important index to reflect the disease severity of colorectal inflammation. The results in Figure 2(E,F) indicated that the colon length was evidently shortened in mice of DSS group (*p* < .01). Nevertheless, oral administration with various therapeutic reagents ameliorated the colon shortening with varying degrees. In addition to the BRH-SMEDDS group, there was no statistical significance in the enhancing effect among other therapeutic groups as compared to BR-suspension group. The therapeutic action of BRH-SMEDDS (1 mg/kg) was more pronounced than that of SASP (*p* < .01), and BRH-SMEDDS exhibited similar therapeutic activity with AZA ( *p* < .05). It is noteworthy that the enhancing effect of BRH-SMEDDS group was nearly 2-fold stronger than that of BR-suspension, which well corresponded to the finding of the pharmacokinetics studies (relative bioavailability of 188.20%), indicating the corresponding relationship of dose-related effect of BR in the two mediums and the pharmacokinetic behaviors.

#### The histological and morphological examinations

**Figure 3.**
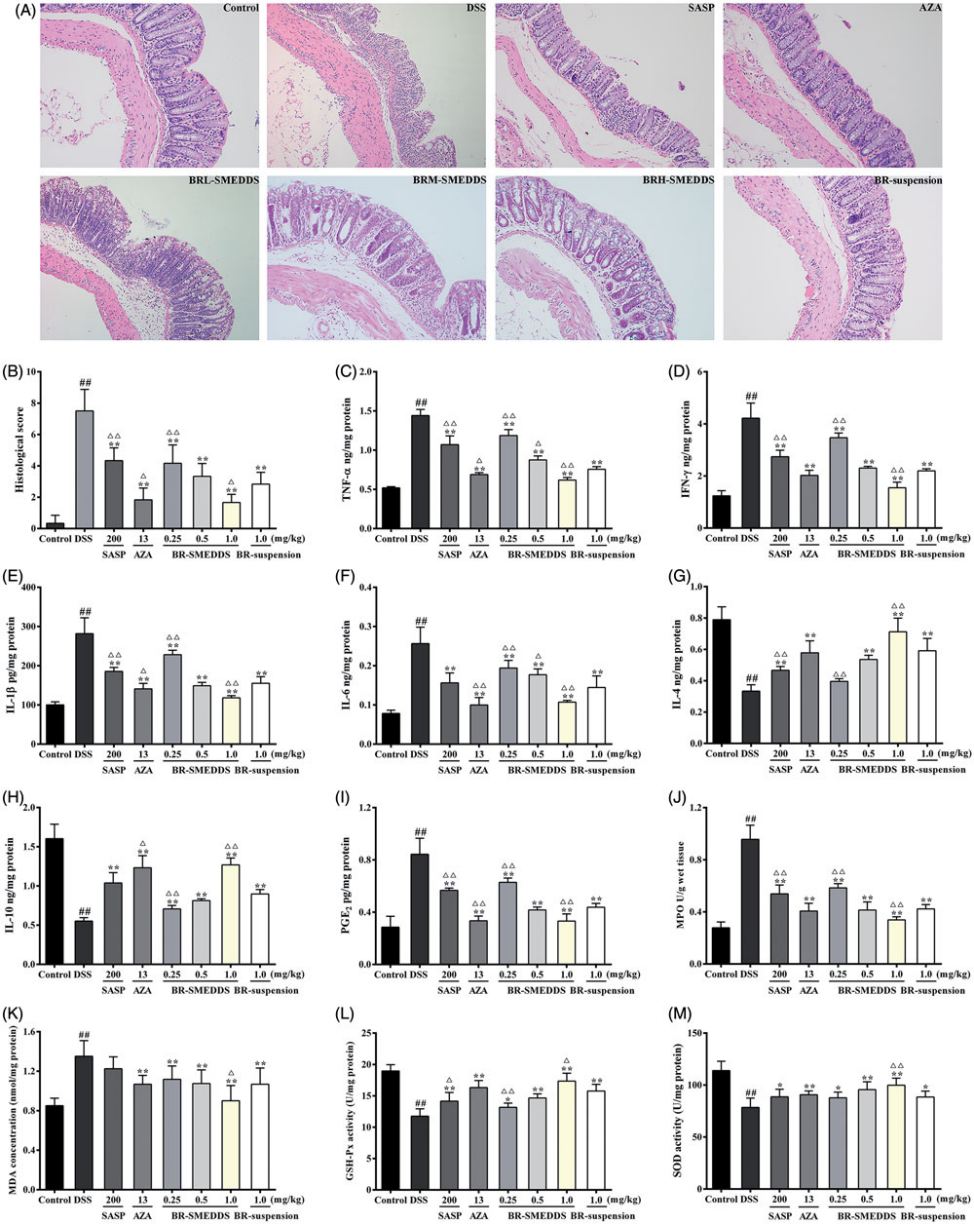
Histomorphological behavior and effects on the production of biochemical cytokines in colorectums. (A) Representative H&E staining slices from colorectal sections, original magnification 200×. (B) Histopathological scores. (C) TNF-α. (D) IFN-γ. (E) IL-1β. (F) IL-6. (G) IL-4. (H) IL-10. (I) PGE_2_. (J) MPO. (K) MDA. (L) GSH-Px. (M) SOD. Data are presented as mean ± SD of six mice in each group. #*p* < .05 and ##*p* < .01 versus control group, *p < .05 and **p < .01 versus DSS group, ^△^ *p* < .05 and ^△△^ *p* < .01 versus BR-suspension group.

The histological and morphological characteristics of the colons were assessed after H&E staining, and the representative results as well as the microscopic scores are presented in Figure 3(A,B). Sections from the control group showed intact surface epithelium, crypt, muscularis mucosa and submucosa. However, the DSS-treated mice exhibited inflammatory cell infiltration, edema, crypt abscesses or loss and extensive submucosal edema, resulting in a high microscopic damage score. On the contrary, treatment with different concentrations of BR in SMEDDS or in suspension and the positive drugs, resulted in progressive restoration, protection of the colon crypt structures and reduction of severe histologic inflammation, therefore drastically lowering the microscopic damage scores. Of note, the most obvious amelioration of inflammation (*p* < .01) was observed in BRH-SMEDDS (1 mg/kg) group, resembling normal mucosa in the control group. And BR-suspension (1 mg/kg) administration to DSS-induced colitis in mice resulted in obvious protection with less severe histologic inflammation, which was similar to the effect of BRM-SMEDDS (0.5 mg/kg) (*p* < .05). And at the same dose of BR (1 mg/kg), the BRH-SMEDDS group was observed to exert more pronounced effect in attenuating these deteriorating pathological alternations than BR-suspension group (*p* < .05). Our results manifested that there was probably a beneficial effect of BR in preventing DSS-induced colitis, and the therapeutic effect was closely associated with the dosage and formulation.

#### Effects on the productions of immune-inflammatory cytokines and PGE_2_ in colorectums

As shown in Figure 3(C–I), the levels of pro-inflammatory cytokines including TNF-α, IFN-γ, IL-1β and IL-6 ascended quickly in DSS-treated mice (*p* < .01) in comparison with those of control group. However, the elevated levels of these pro-inflammatory cytokines induced by DSS were strikingly attenuated by different formulations of BR and positive drugs (*p* < .01). On the other hand, IL-4 and IL-10 were observed to decrease to a low level after DSS induction. While oral administration with different kinds of therapeutic reagents significantly increased the productions of IL-4 and IL-10 (*p* < .01).

Additionally, the DSS-treated colonic level of PGE_2_ was notably increased as compared with that of the control group (*p* < .05). On the contrary, treatments with BR in SMEDDS , in suspension and as well in positive drugs were able to remarkably ameliorate PGE_2_ production as compared with the DSS group (*p* < .01). It was observed that high dose of BR (1 mg/kg) in SMEDDS exhibited relatively stronger efficacy in relieving anomaly of immune-inflammatory cytokines in colorectums, which was more effective than the same dose of BR in suspension (*p* < .01–.05). However, there was no obvious difference between the BR-suspension group (1 mg/kg) and BRM-SMEDDS (0.5 mg/kg) group in regulating the productions of IFN-γ, IL-1 b, IL-4, IL-10 and PGE_2_ (*p* < .05). On the basis of these observations, it was indicated that BR treatment could effectively attenuate the abnormity of immune-inflammatory cytokines, which was intimately correlated to its application dosage and formulation, contributing to the protection from DSS-induced colitis in mice.

#### Effects on oxidative stress in colorectums

As shown in Figure 3(J–M), the MPO and MDA levels in the colon tissues of DSS group were significantly increased, while the SOD and GSH-Px activities in colonic tissues of DSS group were obviously decreased in comparison with those of the control group (*p* < .01). Whereas pretreatment with BR-SMEDDS significantly relieved the oxidative stress, by reducing MPO and MDA contents and enhancing SOD and GSH-Px levels (*p* < .01) in the colon when compared with the DSS group. The oxidative abnormalities in colonic mucosa were observably ameliorated by treatment with BR-SMEDDS in a dose-dependent manner. And there was significant statistical difference (*p* < .01) between the high dose of BR (1 mg/kg) in SMEDDS and suspension in diminishing oxidative stress. BR-suspension exhibited similar therapeutic effect in relieving oxidative damage when compared with BRM-SMEDDS (0.5 mg/kg) or AZA (*p* < .01). Besides, administration with SASP and AZA also resulted in significant increment in SOD and GSH-Px levels (*p* < .01–.05 ), and decreased MPO (*p* < .01) level in parallel to the DSS group, though no statistical significance (*p* < .05) on the MDA content was observed in the SASP group.

#### Effects on the protein expression of TLR4, MyD88 and NF-κB p65 in colorectums

**Figure 4.**
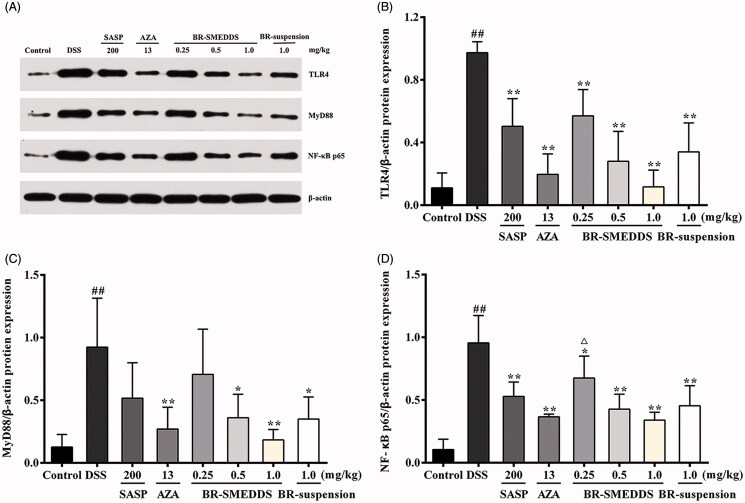
Effects on the protein expression of TLR4, MyD88 and NF-κB p65 in colorectums by Western blotting. (A) Representative Western blotting images of TLR4, MyD88 and NF-κB p65 protein expression in DSS-induced colonic tissues. (B) Changes in the expression level of TLR4 protein. (C) Changes in the expression level of MyD88 protein. (D) Changes in the expression level of NF-κB p65 protein. Data are presented as mean ± SD of three mice in each group. #*p* < .05 and ##*p* < .01 versus control group, *p < .05 and **p < .01 versus DSS group, ^△^ *p* < .05 and ^△△^ *p* < .01 versus BR-suspension group.

The transcriptional factor NF-κB is one of the major proinflammatory signaling pathways involved in UC (Pervin et al., 2015). We subsequently determined the possible involvement of TLR-4, MyD88 and NF- κB protein expression levels in the anti-UC effects of BR-SMEDDS in DSS-induced colitis mice by Western blotting analysis. In this assay, the expression of TLR4, MyD88 and NF-κB p65 total proteins in the colon tissue from colitic group was notably elevated after DSS exposure. Whereas, pretreatment with BR of different dosages in SMEDDS led to significant attenuation of expression of TLR4 in a dose-dependent manner (*p* < .01; Figure 4(B)). MyD88 and NF-κB p65 proteins had no obvious alterations in the presence of low dose of BR-SMEDDS and SASP pretreatment (*p* < .05; Figure 4(C,D)). BR-SMEDDS at the dose of 1 mg/kg resulted in evident reduction of the expression of all the three proteins in mice with colitis (*p* < .01–.05 ). Though BRH-SMEDDS exerted more obvious effect in suppressing the protein expression levels of TLR4, MyD88 and NF-κB p65 in colorectums as compared with the BR-suspension group, the difference did not arrive at statistically significant level.

## Discussion

*Brucea javanica* is traditionally used for the treatment of dysentery, which is also known as UC according to Chinese medicine theory (Han, 2014). Current investigations on *Brucea javanica* have predominantly focused on its anti-cancer properties, however, few efforts have contributed to the exploration of its major traditional application such as dysentery, nor the active component and mechanism of action obligatory for its traditional application.

BR, one of the major bioactive principles of *Brucea javanica*, was demonstrated to exert various bioactivities, such as antimalarial, anti-inflammatory, antineoplastic and anthelmintic activities. Our preliminary experiment has also found that BR could effectively ameliorate DSS-induced UC in mice. In spite of its reputed biological effectiveness, relatively low aqueous solubility and rapid first-pass metabolism after oral administration have badly limited its potential medicinal application. Therefore, it is of vital importance to increase its aqueous solubility and decrease its metabolic clearance simultaneously. SMEDDS has many properties making it appealing as a universal vehicle for the delivery of insoluble lipophilic drugs. Indeed, previous studies have shown that nanoparticulate drug delivery systems were beneficial for prolonging exposure time, increasing the drug efficacy, reducing the side effects, and overcoming the poor bioavailability of the main active components of *Brucea javanica* (Chen et al., 2013). In the present study, pioneering endeavor was devoted to develop SMEDDS nanoformulation containing BR and to evaluate its physicochemical characterizations including solubility, droplet size, and release performance, pharmacokinetic behavior, the potential anti-colitis effect and the potential underlying mechanism.

The droplet size of microemulsion is a critical factor for self-emulsification since it is closely associated with the rate, extent and absorption of drug release. Droplet size and TEM results showed that BR-SMEDDS would self-emulsify to form nanosized microemulsion droplets when diluted with distilled water. At the same time, low PDI reflects the uniformity of particle size in the microemulsion. The closer the PDI value is to zero, the more homogeneous the droplets are (Zhao et al., 2013b). Stability of nanoparticles depends in part on the surface zeta potential, a parameter which gives the magnitude of the electrostatic repulsive interactions between particles (Doane et al., 2012). It is generally accepted that a higher value of zeta potential hinders the probability of coalescence and thereby maintains homogeneity of droplet size (Yuan et al., 2014). In the present work, the BR-SMEDDS formulation obtained exhibited a higher negative average zeta potential, which complied with the required zeta potential prerequisite for a stable microemulsion. Stability tests revealed that different pH mediums had no marked effect on the particle size distribution of BR-SMEDDS, indicating acceptable stability in the gastrointestinal tract and therefore potential enhancement in absorption provided by the nanosized structure. The zeta potential of BR-SMEDDS was distinctly different in the simulated gastric fluid. The reason was assumed to be attributed to the existence of ion in the solution resulting in the zeta potential change (Li et al., 2014a). However, further in-depth investigation is warranted to unravel the definitive mechanism.

The bioavailability behavior of BR-SMEDDS formulation was also investigated in our study. From the result, the *T*_max_ of BR-SMEDDS was relatively diminished as compared to the free drug suspension. This observation might indicate that the BR-SMEDDS self-emulsification process could improve the release and absorption of BR in the gastrointestinal tract, which was in accord with previous report (Dangre et al., 2016). The *t*_1/2_ and MRT_0–24_ were significantly prolonged as compared with the BR-suspension, suggesting that BR in the SMEDDS might circulate for a longer time within the body. In addition, the increase in the bioavailability might be owing to the predominantly improved solubility of BR by microemulsion, which maintained BR as the soluble form during the gastrointestinal dilution and permeation process (Ting et al., 2014). Another reason for oral bioavailability improvement was possibly the high contents of the surfactant acting as an absorption enhancer (Kawakami et al., 2002). In addition, oil phase in the formulation promoted transport of the lipophilic drug, avoiding being metabolized by the liver and therefore enhancing drug absorption and bioavailability (Dangre et al., 2016). However, the exact mechanism is merited for further investigation.

In the present study, the BR-SMEDDS was prepared to evaluate the efficacy of anti-colitis activity in parallel to the aqueous suspension. We employed an *in vivo* model of colitis induced by DSS in mice, which was widely used as a validated model of chemically induced colitis that closely resembled UC in human (Chen et al., 2015a). In our study, the clinical symptoms of DSS-induced model including weight loss, diarrhea, fecal blood, ulcerations, infiltrations with granulocytes, and shortened colons were observed. It was found that the therapeutic effect of BR-SMEDDS was notable, resulting in weight regain as well as decreased intestinal bleeding as compared with the DSS-induced mice. Besides, the DAI scores were markedly attenuated and the colon length was noticeably recovered by BR-SMEDDS in a dose-dependent manner. The therapeutic effect of BRH-SMEDDS was shown to be superior to that of BR-suspension and SASP, and was similar to that of AZA, with much smaller dosage. Furthermore, BR-SMEDDS protected against both the infiltration of inflammatory cells and the mucosal damage, leading to a significant reduction of histopathological damage. Under the same dosage, the BR-SMEDDS exhibited more evident therapeutic effect compared with BR-suspension, indicating the SMEDDS formulation exerted improved efficacy in the anti-colitis effect of BR. Meanwhile, treatment with BR-suspension had an analogous effect to the lower dose of BR in SMEDDS, which well corresponded with the results of the pharmacokinetic study, indicating the enhanced therapeutic efficacy of BR against UC might be intimately related to the improved pharmacokinetic property by SMEDDS.

It has been generally accepted that various cytokines are considered as crucial signals in the intestinal immune system (Pandurangan et al., 2015). Cytokines control different aspects of the inflammatory response in UC. The imbalance between pro- and anti-inflammatory cytokines disrupts ease of inflammation and further leads to an elongation of the disease (Jang et al., 2016). TNF-α, IFN-γ, IL-1β, and IL-6 have long been recognized as key pro-inflammatory mediators causing the intestinal mucosal impairment and resulting in inflammation (Niu et al., 2016; Yang et al., 2016). The cytokines such as TNF-α and IL-1β are released with subsequently enhanced synthesis of PGE_2_ and exacerbation of tissue damage (Nagib et al., 2013). Blocking the production of these cytokines can ameliorate the inflammation in colitis mice. The cytokines IL-4 and IL-10, also known to have strong immunosuppressive and immunoregulatory factors, could effectively inhibit the pro-inflammatory cytokine synthesis and antigen presentation, causing alleviation of inflammatory responses (Socca et al., 2013; Xiong et al., 2013). Low expression level in these cytokines promoted the inflammatory reaction of intestine mucosa and developed spontaneous severe UC (Zhou et al., 2014). In our experiment, BR-SMEDDS treatment effectively inhibited the levels of pro-inflammatory cytokines and PGE_2_, and substantially promoted the production of the immunoregulatory mediators IL-4 and IL-10. High-dose BR-SMEDDS seemed to exert more pronounced anti-inflammatory effect than the positive drugs and the aqueous solution. These results were congruent with the dose-related decrease of infiltration of inflammatory cells and attenuation of the mucosal damage in BR-SMEDDS treatment group.

Oxidative stress is another crucial factor contributing to the pathogenesis of UC and inflammatory process. It is known to damage cellular macromolecules such as DNA, lipids, and proteins, which could aggravate free radical chain reactions, disrupt the integrity of the intestinal mucosal barrier, and activate inflammatory mediators (de Faria et al., 2011; Pandurangan et al., 2015). MPO is an enzyme abundantly found in neutrophils which could trigger a variety of inflammatory diseases (Chen et al., 2015b; Rashidian et al., 2016). At the same time, MDA is considered as a byproduct of lipid peroxidation, and increased MDA level causes the cross-linking of protein and nucleic acid molecules and cell toxicity (Zhao et al., 2013a). SOD and GSH-Px are the major antioxidant enzymes in organisms. SOD is a key enzyme which inactivates superoxide ion by transforming it into H_2_O_2_, a more stable metabolite, and the H_2_O_2_ is further converted to water by catalase or GSH-Px. GSH-Px is an antioxidant enzyme that helps in scavenging and inactivation of free radicals thereby protecting the body against oxidative stress (Kannan & Guruvayoorappan, 2013). MDA is an essential co-factor of GSH-Px and SOD and plays a significant oxidative role by binding to the active site of GSH-Px (Zhao et al., 2013a). Therefore, measurement of enzymatic activities can be interpreted as a manifestation for assessing acute intestinal inflammation. Our study demonstrated that the beneficial effect of BR-SMEDDS might be intimately associated with the enhancement of antioxidant enzymes including SOD and GSH-Px, as well as dose-dependent amelioration of MPO and MDA levels. The results obtained clearly indicated that the BR-SMEDDS formulation might exert its anti-colitis effect, at least in part, via regulation of oxidative stress biomarkers. Together, the favorable regulation of anti-oxidative and anti-inflammatory status might contribute to the amelioration of histopathological deterioration of BR-SMEDDS treatment.

TLR4/NF-κB pathway, is one of the important pathways that involve a series of proteins and cytokines, which mediates inflammatory responses and plays a key role in the pathogenesis of UC. TLR4, a member of the Toll-like family of proteins recognizes lipopolysaccharide and mediates transmembrane signal transduction. After interaction with lipopolysaccharide, TLR4 is activated followed by the recruitment of the MyD88, a major adaptor molecule essential for TLR signaling. This in turn triggers a signaling cascade which results in the activation of the downstream NF-κB (He et al., 2016). NF-κB is an important transcription factor during immune response regulation. Activation of NF-κB could increase the induction of pro-inflammatory cytokines and growth factors eventually resulting in the development of colitis, among which, p65 is a major functional subunit in the NF-κB family (Xing et al., 2013; Zhang et al., 2016a). In the present study, treatment with BR-SMEDDS or BR aqueous solution caused significant attenuation of TLR4, MyD88 and NF-κB p65 expression in the colon tissue. This result proposed that inhibition of the TLR4-linked NF-κB signaling pathway might play a crucial role in the protection of BR against experimental UC. Of note, no significant difference in the protein expression levels of TLR4, MyD88 and NF-κB p65 between BR-suspension group and BR-SMEDDS groups was observed. This might indicate other pathways might also play an important role in the anti-UC effect of BR. However, further investigation is merited to illuminate the more precise mechanism.

Our results strengthened our previous findings to indicate the potential of active component of *Brucea javanica* as a possible anti-UC agent. However, in-depth definitive studies are merited to unveil in greater detail its mechanism. Also, long-term safety evaluation and more detailed pharmacokinetics should be investigated prior to possible future clinical trials. Some of these initiatives are already underway in our laboratory. These enlightenments in the future should provide novel dimension for the potential application of BR-SMEDDS as a naturally-occurring agent for the treatment of UC.

## Conclusion

Taken together, our findings demonstrated for the first time that administration of BR could effectively attenuate colonic inflammation in mice, at least partially, via favorable regulation of anti-oxidative and anti-inflammatory status and inhibition of the TLR4-linked NF-κB signaling pathway. BR in SMEDDS achieved superior therapeutic effect as compared to its aqueous suspension and SASP, and BRH-SMEDDS showed similar anti-UC effect to AZA, but with much smaller dose. The enhanced therapeutic efficacy of BR against UC might be intimately associated with the improved pharmacokinetic property of BR by SMEDDS. The studies indicated that the developed BR-SMEDDS possessed good clarity, excellent stability, high entrapment efficiency, improved oral bioavailability, and enhanced anti-colitis efficiency as compared with BR suspension. The nanoparticulate system might represent a promising drug carrier for improving the physiochemical properties and enhancing the therapeutic efficacy of BR. Therefore, BR-SMEDDS might have the potential to be further developed into a promising anti-colitis agent.
